# Practical Barriers and Ethical Challenges in Genetic Data Sharing

**DOI:** 10.3390/ijerph110808383

**Published:** 2014-08-15

**Authors:** Claire L. Simpson, Aaron J. Goldenberg, Rob Culverhouse, Denise Daley, Robert P. Igo, Gail P. Jarvik, Diptasri M. Mandal, Deborah Mascalzoni, Courtney Gray Montgomery, Brandon L. Pierce, Rosemarie Plaetke, Sanjay Shete, Katrina A. B. Goddard, Catherine M. Stein

**Affiliations:** 1 Computational and Statistical Genomics Branch, National Human Genome Research Institute, Baltimore, MD 21224, USA; 2 Department of Bioethics, Case Western Reserve University, Cleveland, OH 44106, USA; E-Mail: aaron.goldenberg@case.edu; 3 Department of Internal Medicine, Washington University in St. Louis, St. Louis, MO 63110, USA; E-Mail: rculverh@dom.wustl.edu; 4 Department of Medicine, University of British Columbia, Vancouver, BC V6Z 1Y6, Canada; E-Mail: denise.daley@hli.ubc.ca; 5 Department of Epidemiology & Biostatistics, Case Western Reserve University, Cleveland, OH 44106, USA; E-Mails: rpi@case.edu (R.P.I.); cmj7@case.edu (C.M.S.); 6 Departments of Medicine (Medical Genetics) and Genome Sciences, University of Washington, Seattle, WA 98195, USA; E-Mail: gjarvik@medicine.washington.edu; 7 Department of Genetics, Louisiana State University Health Sciences Center, New Orleans, LA 70112, USA; E-Mail: dmanda@lsuhsc.edu; 8 EURAC Institute of Genetic Medicine, Bolzano 39100, Italy; E-Mail: deborah.mascalzoni@eurac.edu; 9 Oklahoma Medical Research Foundation, Oklahoma City, OK 73104, USA; E-Mail: courtney-montgomery@omrf.org; 10 Department of Health Studies, University of Chicago, Chicago, IL 60637, USA; E-Mail: bpierce@health.bsd.uchicago.edu; 11 Department of Medical Biometry and Epidemiology, University Medical Center Hamburg-Eppendorf, Martinistrasse 52, 20246 Hamburg, Germany; E-Mail: rplaetke@alaska.edu; 12 UT MD Anderson Cancer Center, Houston, TX 77030, USA; E-Mail: sshete@mdanderson.org; 13 Center for Health Research, Kaiser Permanente Northwest, Portland, OR 97227, USA; E-Mail: Katrina.AB.Goddard@kpchr.org

**Keywords:** data sharing, identifiability, GWAS, ELSI, ethics, publication embargo, collaboration

## Abstract

The underlying ethos of dbGaP is that access to these data by secondary data analysts facilitates advancement of science. NIH has required that genome-wide association study data be deposited in the Database of Genotypes and Phenotypes (dbGaP) since 2003. In 2013, a proposed updated policy extended this requirement to next-generation sequencing data. However, recent literature and anecdotal reports suggest lingering logistical and ethical concerns about subject identifiability, informed consent, publication embargo enforcement, and difficulty in accessing dbGaP data. We surveyed the International Genetic Epidemiology Society (IGES) membership about their experiences. One hundred and seventy five (175) individuals completed the survey, a response rate of 27%. Of respondents who received data from dbGaP (43%), only 32% perceived the application process as easy but most (75%) received data within five months. Remaining challenges include difficulty in identifying an institutional signing official and an overlong application process. Only 24% of respondents had contributed data to dbGaP. Of these, 31% reported local IRB restrictions on data release; an additional 15% had to reconsent study participants before depositing data. The majority of respondents (56%) disagreed that the publication embargo period was sufficient. In response, we recommend longer embargo periods and use of varied data-sharing models rather than a one-size-fits-all approach.

## 1. Introduction

In 2003, the National Institutes of Health (NIH) implemented a data sharing policy requiring data obtained in NIH-supported or conducted genome-wide association studies (GWAS) to be posted to the database of Genotypes and Phenotypes (dbGaP) or a similar database (NOT-OD-07-088,) [1]. In 2013, the policy was updated to cover the technological advances in next-generation sequencing so that whole-exome and whole-genome sequencing studies would also be required to share their data through dbGaP or similar databases. There are several potential advantages of such a data sharing policy. First, open access to such complex data reduces barriers and may more rapidly advance science and creativity, leading to novel discoveries [2]. Second, data sharing maximizes the return on the public investment in generating such data, an especially pertinent consideration if one believes that society owns publicly funded data [3].

These advantages are counter-balanced by practical and ethical concerns in the implementation of this data sharing policy, and in some cases the NIH has already responded to address issues raised by the research community. One issue is that specific individuals can be identified based on genomic data when combined with other publicly available data resources [4,5,6]. Another study showed that a surname can be linked with an individual’s genomic data using genealogical databases for descendants of the Latter-Day Saints founders [7]. Indeed, the U.S. Department of Health and Human Services has proposed to update the common rule to specifically define biospecimens and genetic data as identifiable (docket ID number HHS-OPHS-2011-0005; Advance Notice of Proposed Rulemaking (ANPRM) regarding Human Subject Research Protections). A second issue is that an individuals’ risk for a specific disease can still be inferred even if known risk allele data is removed. For example, based on the linkage disequilibrium structure of APOE, as well as available HapMap data, one could accurately predict Alzheimer’s disease risk for an individual even after removal of the individual’s APOE genotypes from the analysis [8]. Based on these findings, allele frequencies and other summarized data were no longer publicly posted on the NIH site. More recently, it was shown that a number of individuals from the Center for Study of Human Polymorphisms (CEPH) family collection whose genomes were sequenced as part of the 1000 Genomes project could be identified [9].

Other concerns with the implementation of the data sharing policy have not yet been fully addressed. First, although the policy allows waivers to the requirement for submission of data to dbGaP, in practice the implementation of this policy occurs at the institute level, which could allow inconsistency in how waivers are managed or approved. Much of the need for waivers stems from the re-use of DNA samples for genetic studies that were collected prior to the discussion of identifiability and shared databases; thus, appropriate language about possible risks or benefits to the donors/participants was never included in informed consent forms. Second, a subject’s option to withdraw from a study is of limited effectiveness once the data is shared in public, as that data has already been distributed, often cannot be recalled, and therefore may be used for further analysis even against the will of participants [10]. Third, a publication embargo period was established on many, but not all, data submitted to dbGaP to recognize the intellectual contributions of the data submitters in establishing the data resource and provide them with the first opportunity for publication; however, a violation of the publication embargo has already occurred [11]. In that case, the authors were reprimanded by both their institution and the NIH, and although the publication was officially retracted by the journal, it remains available online. Few studies have explored the perceived practical and ethical concerns of researchers regarding data sharing from GWAS, and whether those concerns represent significant barriers to the success of dbGaP and other methods of data sharing. To that end, we conducted a survey of the membership of the International Genetic Epidemiology Society (IGES) to explore the experiences of researchers with dbGaP. IGES is a scientific society concerned with “the study of genetic components in complex biological phenomena” [12]. IGES members include geneticists, epidemiologists, statisticians, mathematicians, biologists, related biomedical researchers and students interested in research on the genetic basis of diseases, complex traits, and their risk factors. This survey population may provide generalizable insights for several reasons. First, the IGES membership is involved in human genetic research and includes scientists who have contributed data to dbGaP. Second, IGES members, especially those who are developing novel statistical methods and genetic epidemiological techniques, are among the researchers most interested in obtaining data from dbGaP. Finally, the practical barriers and perceived ethical challenges experienced by IGES members with dbGaP use for genome-wide association (GWA) data have relevance for future sharing of whole exome (WES) and whole genome sequencing (WGS) data.

## 2. Experimental Section

### 2.1. Survey Design and Implementation

Members of the IGES Ethical, Legal, and Social Issues (ELSI) committee designed the survey instrument (supplemental materials). The committee is a multidisciplinary group generally interested in current ELSI issues as they relate to genetic epidemiologists. Committee members come from a variety of backgrounds and have varied research interests ranging from biology, medicine, statistical genetics, and epidemiology, to bio-ethical theory and law. Questions, both multiple choice and free-text, covered domains including demographics, past application for research funding, contribution of data to dbGaP, access of data through dbGaP and other similar databases, and avoidance of the data sharing policy. We implemented the survey, containing 44 questions, on SurveyMonkey^®^ [13]. Members of the IGES ELSI committee pilot tested the survey to check the skip patterns to the questions and ease of use; however, we did not pilot test the survey with external reviewers to assess the readability or comprehension of the survey questions. All IGES members were invited to complete the survey by email through the IGES membership listserver. We sent two reminder emails, as well as a post to the “Friends of IGES” Facebook page. No incentive was given for participation in the survey, and no identifying information (e.g., IP address) was obtained from the survey participants. The survey was open for response from October to November of 2010.

### 2.2. Data Analysis

We downloaded a dataset containing all participant response data from SurveyMonkey as an Excel file. We computed descriptive statistics and statistical analyses using SPSS version 19 (IBM Corp. Armonk, NY, USA)). Percentages are reported based on the number of valid responses for each question. Analyses of categorical data were conducted using chi-square statistics. Eight questions were open-ended; responses for those could be entered into a box that could contain a brief paragraph. Eleven questions provided an “other” category as a response; the respondent could then specify an alternative response. We analyzed data from open-ended questions by looking for common themes. Responses were first separated into the two major thematic categories that corresponded to either common practical or ethical concerns. These responses were then further analyzed to identify more specific areas of concern.

## 3. Results and Discussion

A total of 187 individuals started the survey; 175 out of 643 IGES members completed it (Table 1), resulting in a response rate of 27%. Most of the survey respondents (77%) were “Regular” (*i.e.*, not student) IGES members. The distribution of survey respondents by continent was roughly equivalent to the overall distribution of IGES membership (Table 1).

Most of the survey respondents work in academic or university environments (Table 2). There was a fairly uniform distribution of respondents across time spent in their current position, with the exception that fewer people had spent 11 to 15 years at their current position. Most of the faculty respondents had applied to the NIH for research funding (83%), regardless of whether they worked in North America (Table 2).

**Table 1:** Demographics of survey respondents compared to overall IGES membership.

	Survey Respondents ^a^	IGES Overall Membership ^a^
Students	33 (18%)	146 (23%)
Regular	147 (82%)	497 (77%)
**Total**	**175**	**643**
Continent / region		
United States	138 (74%) ^b^	428 (67%)
Canada		37 (6%)
Europe	39 (21%)	123 (19%)
Australia, New Zealand, and Other	9 (5%)	55 (8%)

^a^ Data shown as absolute frequencies (% of total). ^b^ These two categories are combined because the survey only asked whether respondents worked in North America, so that data were not identifiable.

**Table 2:** Survey respondents’ position characteristics.

Characteristic	N (%)
**Type of organization**	
Academic or university	129 (72%)
Government agency	17 (9%)
Hospital	11 (6%)
Research Institute	22 (12%)
Industry	1 (0.5%)
**Length of time in current position**	
< 3 years	52 (29%)
3 to 5 years	37 (21%)
6 to 10 years	41 (23%)
11 to 15 years	15 (8%)
> 15 years	35 (19%)

### 3.1. Data Access

Of all respondents, 43% (N = 80) reported accessing data from dbGaP at least once (Table 3). When asked, “How long did it take to complete the application and receive the data the first time you applied?”, 37.5% (N = 30) stated it took less than 3 months, and an additional 37.5% (N = 30) indicated that it took 3–4 months. Among those who reported applying for data more than once (N = 30), responses to the question, “How long did it take to complete the application and receive the data the most recent time you applied?” did not differ noticeably. When asked how much they agreed/disagreed that the process of requesting data from dbGaP was easy, many respondents (32%) agreed. The duration of time it took to complete application and receive the data was associated with whether the respondent agreed that the dbGaP application process was perceived as easy (*p* = 0.015).

We also asked what sort of ethics board approval, if any, was required when accessing dbGaP data. Sixteen percent (N = 13) of individuals reported full ethics board approval, 38% (N = 30) reported an expedited review, 20% (N = 16) reported that their institution waived the requirement for ethics board approval, and 15% (N = 12) reported that their institution did not require ethics board approval (Table 4). Thus, ethics board approval requirements were widely variable across institutions; this did not differ significantly by the type of institution (*p* = 0.153) or by continent (*p* = 0.312) (data not shown). The type of IRB approval needed was not associated with whether the respondent agreed that the dbGaP application process was perceived as easy (*p* = 0.239).

**Table 3:** Attitudes towards dbGaP access issues ^a,b^.

	Strongly Agree	Agree	Disagree	Strongly Disagree	Undecided / Don’t Know	Missing / No Response
The process for requesting data from dbGaP was easy	2 (3)	23 (29)	25 (31)	14 (18)	9 (11)	7 (9)
It was easy to find a signing official	29 (36)	23 (29)	7 (9)	7 (9)	1 (1)	13 (16)

^a^ Numbers are absolute frequencies (%); ^b^ Only individuals who reported requesting data from dbGaP responded to this question (N = 80).

**Table 4:** IRB application experience for individuals requesting dbGaP data.

Response	N (%) ^a^
Yes, I had to go through full-board review	13 (16)
Yes, I had to go through an expedited IRB (or equivalent) review	30 (38)
No, the IRB (or equivalent) waived the requirement for approval, or the study was considered exempt	16 (20)
No, my Institution does not require me to apply for IRB (or equivalent) approval to obtain and analyze data from dbGaP	12 (15)
*Missing / no response*	9 (11)

^a^ Data shown as (absolute) frequencies (%), N = 80.

An “institutional official” needs to sign a researcher’s application for dbGaP data, a concept that may not be well-defined for organizations that do not receive funding from NIH. Most individuals (65%) either agreed or strongly agreed that it was easy to identify a signing official. However, the title of the signing official varied widely. In the free-text field where respondents listed the title of the signing official, 12 individuals reported a title of someone in a research administration office, six individuals reported the title of an academic head or scientific director, three individuals reported a “director” position that did not appear to fit into a research office or academic head, and two individuals reported getting the signature of the president of the university. The ease of identifying a signing official differed significantly by type of institution, with more people in research institutes reporting difficulty with identifying the signing official (*p* = 0.003, data not shown). This issue was also associated with continent, with more Europeans reporting problems than North Americans (*p* = 0.029, data not shown); five European respondents (36%) either agreed or strongly agreed that it was difficult to find a signing official, and four individuals at research institutes (44%) also agreed or strongly agreed it was difficult to find a signing official. The ease of identifying a signing official was associated with whether the respondent agreed that the dbGaP application process was perceived as easy (*p* = 0.005).

Only 19% of individuals (N = 15) reported contacting the original data contributors regarding questions about the data.

### 3.2. Data Contributors

Twenty-four percent (N = 45) of all respondents reported being the principal investigator or co-investigator on a study that contributed data to dbGaP. We note that dbGaP submission requires the local IRB to certify consistency with laws and regulations, which can vary by state or country, and with the content necessary to be provided in informed consent documents, which can vary by study as well as by state or country. Of the respondents who contributed data to dbGaP, 31% (N = 14) reported local IRB restrictions on data release, and 31% (N = 14) reported that they had to reconsent study participants (seven individuals answered “yes” to both questions). Of the individuals who did reconsent their study participants, at least 32% did not inform them of the risk of being identified from genotype or phenotype data (Table 5).

**Table 5:** Information about depositing data to dbGaP from 45 responders.

Questions and Responses	N (%) ^a^
*For how many studies have you deposited data into dbGaP?*	
1	28 (62)
2	12 (27)
3	1 (2)
5	2 (4)
Missing / no response	2 (4)
*For any of the studies that you contributed to dbGaP, did your IRB (or equivalent) put any restrictions on data release?*	
Yes	14 (31)
No	21 (47)
Not sure	9 (20)
Missing / no response	1 (2)
*For any of the studies that you contributed to dbGaP, did you reconsent the research participants in order to deposit the data on dbGaP*	
Yes	14 (31)
No	24 (53)
Not sure	6 (13)
Missing / no response	1 (2)
*When you reconsented individuals, did you inform individuals about the potential of being identified from their genotype or phenotype data?^a^*	
Yes	6 (43)
No	3 (21)
Not sure	5 (36)
*How much do you agree with the following statement: The embargo period was sufficient to perform the analyses that I wanted to do?*	
Strongly agree	1 (2)
Agree	9 (20)
Disagree	12 (27)
Strongly disagree	13 (29)
Undecided	6 (13)
Too early to determine	3 (7)
Missing / no response	1 (2)

^a^ Denominator for this question is N=14, the number of individuals who reconsented their subjects; ^b^ Data shown as (absolute) frequencies (%)

While seven individuals requested a waiver from submitting data to dbGaP, three of these individuals did not get the waiver granted. The four successful requests for waivers included the following justifications: a foreign study population (N = 1), “no consent” or “not able to reconsent” (N = 2), and “sensitive topic” (N = 1). The three unsuccessful waiver requests provided justifications that included “would require reconsent” (N = 2) and “participating American Indian tribes would not agree to broad data sharing” (N = 1).

Our final question for data contributors was whether the publication embargo period was sufficient to perform the desired analyses. The majority of respondents (56%) either disagreed or strongly disagreed that the embargo period was sufficient (Table 5). Two individuals reported in a free-text field that data they had submitted to dbGaP, had been downloaded and analyzed by another group and the findings published before the embargo period was over.

### 3.3. Alternatives to Depositing Data in dbGaP

Next, we asked a series of questions to get a sense of whether the current data sharing policy limits opportunities for research, and, if so, what are the perceived barriers. Eleven percent of all respondents (N = 21) reported applying for funding elsewhere to avoid the NIH data sharing policy. Fourteen percent (N = 26) of individuals decided not to apply for NIH funding specifically because of the data sharing policy. Fourteen people answered “yes” to both questions. Several reasons were given for this (Table 6). The most prevalent response was that the consent form or local IRB would not allow this type of data sharing (N = 16), or it would be “not legally possible because of requirements in country” (N = 12); six people responded that they “do not trust the system of data sharing”.

**Table 6:** Reasons given for not applying for NIH funding because of the data sharing policy.

Reason	N ^*^
It would not be legally possible to deposit the data in dbGaP according to country’s (or institutional) requirements	13
The procedure was too complicated	3
The consent form would not allow broad data sharing	16
Did not trust system of data sharing	6
The data sharing policy is “not in agreement with my personal ethical opinions”	1
Subjects deceased and cannot be reconsented	1
Inability to require IRB approval by data requestors	1
Vulnerable population	1
Data collected from other countries	1
Social responsibility	1

^*^ Respondents may have selected more than one relevant response, so only absolute frequencies are shown.

### 3.4. Future Implications

We asked whether respondents foresee additional problems with depositing/sharing WGS data; only 32% (N = 60) agreed. Respondents were then given the opportunity to describe their own perceptions of these specific problems through an open-ended question. These qualitative responses are not meant to be generalized or representative, but do provide some insight into the types of concerns researchers may have about interfacing with dbGaP and other large genomic databases. Responses to this question fell into two primary thematic categories: practical barriers associated with the management and “usability” of the database, and ethical concerns regarding contributing data and the future use of DNA and RNA sequence data. However, within each of these categories, respondents were varied in their actual concerns.

First, with regard to the practical challenges of dbGaP, some respondents were skeptical of the sustainability of dbGaP itself given concerns about the ability of the system to store and manage such large datasets while making them easily accessible to researchers. Others expressed more specific concerns about the ability of researchers to interface with dbGaP for purposes of both transferring data to the database and enabling researchers to access the data for future research. One researcher wrote that (s)he perceived “data transferral with these large datasets will be highly problematic” while another voiced concerns over the amount of “pre-processing and standardization” needed for large datasets. Additionally, respondents expressed concerns regarding the utilization of data in dbGaP for future studies, including a number of researchers that indicated apprehension about the time required to download larger datasets and others who felt that the user interface was hard to use or “cumbersome”. Lastly, a few respondents said that they would question the quality of raw DNA or RNA sequence data from dbGaP in light of their concerns about the ability of dbGaP to effectively manage and usefully transfer to researchers the large volume of information associated with sequence data.

In addition to these practical issues, respondents identified ethical and social implications of contributing and using data associated with dbGaP. Three interconnected concerns were mentioned: (i) the problem of increased identifiability of study participants, (ii) the impact of increased identifiability on the consent process, and (iii) potential difficulties in subject recruitment due to (i) and (ii).

The problem of identifiability is amplified by the ability to mine DNA sequence data for “rare variants”, potentially making an individual or families more identifiable. One respondent thought the contribution of sequence data to dbGaP would compromise the ability of researchers to assure the privacy and confidentiality of study participants, while others felt that participants may worry about their genetic data being used for the identification of deleterious mutations without their knowledge or permission.

An increased risk of identifiability (and concomitant loss of confidentiality) can be expected to have an impact on the consent process. One respondent asked, “How is the consenting of patients for this type of data release going to be handled? Will a general data release statement be sufficient as it was for SNPs? How do we define informed consent?” Another individual discussed fears that contributing whole genome or exome sequence data to dbGaP presents “deeper issues of identification” and raises questions as to “whether we can truly adequately consent people for this type of data sharing”.

Lastly, one researcher expressed a further worry that requirements to contribute data might hurt subject recruitment due to a “possible reluctance of some to participate in studies”. If the sharing requirement for sequence data has a chilling effect on subject recruitment, it would undermine the potential benefits expected from the policy.

## 4. Conclusions

In summary, many respondents to our survey (34%) agreed that they perceive the application process for dbGaP data access as easy and 75% were able to receive data in less than five months. The considerable effort put into the development of the dbGaP system [11] has resulted in a few remaining challenges. These opportunities for improvement of the system, including simplifying the approach to identify a “signing official” and management of large datafiles, are modest when compared with the challenges of initiating new data collection, which can take years. On the other hand, researchers who contribute data to dbGaP face many challenges, primarily local IRB restrictions, the need to reconsent individuals and inform them about potential risks of identifiability, and the short length of the publication embargo period. Significant opportunity for improvement in the implementation of the NIH data sharing policy is still possible from the perspective of data contributors. Further, a mixed approach to data sharing models—including consortiums and workshops—may also alleviate some of these challenges.

### 4.1. Issues for dbGaP Access and Use

The race to publish on one’s own data as it relates to the publication embargo remains an issue. In our relatively small dataset, two individuals reported that data recipients from dbGaP published before the embargo period was over. As stated by Kaye *et al.* [2], “no one wants to be part of a system in which they feel that someone else can take advantage of their contributions.” Furthermore, Kaye *et al.* [2] argue that working against deadlines is not consistent with a productive climate. On the other hand, others argue that without a deadline, researchers hold their data indefinitely without analyzing and publishing, so a deadline is a strong motivating factor. We recommend that the publication embargo policy be reviewed to enable more time for the data contributors to analyze and publish on their own data, which would provide greater protection and recognition of the intellectual contributions of the data contributors and their expertise with the dataset. Additionally, we recommend that enforcing the publication embargo be a shared responsibility between data requestors and others—journal reviewers, journal editors, and the NIH—who can find proactive solutions to identify potential violations before they occur.

In our study, a minority of dbGaP users (<20%) contacted the data collectors, though this was encouraged by the coordinators of dbGaP, as evidenced by the prominent publication of contributors’ contact information. Krawczak *et al.* [14] point out that the bottleneck in generating high quality genetic epidemiological data is mostly in the recruitment and phenotyping of subjects, not in the genotyping. Those who collected the data may be aware of subtleties that can improve analyses or interpretation. Thus, we encourage increased interaction with the data collectors and formal recognition of their efforts. Also, we encourage data collectors to be responsive to the collaborative attempts of secondary data analysts. Depending on the extent of collaboration with the data collectors, recognition of their efforts may be made via co-authorship or in the acknowledgments section of the paper. A recent editorial published in *Nature Genetics* also suggested the use of citable data management plans as another method to acknowledge the data collectors [15].

### 4.2. Ethical Implications for Access and Contribution

We observed a great deal of variability in local IRB requirements for receiving dbGaP data. One possibility is that we observe this variation because there are different requirements for ethics board approval depending on the dbGaP dataset, and it is a limitation of our survey that we did not distinguish which dbGaP datasets the survey respondents had requested. However, this variation among respondents regarding their IRB requirements for dbGaP access may be concerning for contributors to dbGaP, because it may imply variability among IRBs in the understanding of genetic databases and the implications for human subject protection. As recently noted by OHRP in the Advanced Notice of Proposed Rule Making (ANPRM) for revision to the Common Rule [16], this variation is a broad concern, and is not limited to genetic research. McGuire and Gibbs [17] state that, in their experience, the consent process for most disease-specific genetic research is not protective for broad genomic data sharing because privacy risks are not stated. McWilliams *et al.* [18] found that IRB review of genetic epidemiology studies was inconsistent across local IRBs. More recently, Lemke *et al.* [19] found wide diversity among IRB members in the specific ethical implications inherent in genetic data, such as the risk of identifiability, the need for reconsent, clarity in NIH data sharing guidelines, likelihood of individuals being identified from genetic data, and likeliness of harm. The issues of re-consent and warning participants of the risks of identifiability are challenging because these risks are unknown. As pointed out by Kaye *et al.* [2], ethics committees cannot exert their mandates on recipients of data from other institutions. A potential solution for dbGaP is to require data requestors to obtain IRB approval from the IRB where the data were collected through Data Use Limitations [20].

### 4.3. The Consortium Model

A complimentary model to public data sharing, which has been quite successful, is the formation of large consortia in which data from different projects are combined, and a variety of researchers with different expertise in aspects of phenotyping and methodology collaborate to jointly analyze their data. The consortium data sharing model may be particularly advantageous in situations where potential data contributors experience limitations because of local IRB restrictions or a need to reconsent study participants. Often, data are meta-analyzed in order to gain statistical power, and numerous consortia have published on novel loci identified using this model of data sharing. Examples include the CHARGE consortium [21], the CARe consortium [22,23], the Alzheimer Disease Genetics Consortium (ADGC) [24], CGASP [25], other smoking consortia [26,27], LLAS1 and LLAS2 [28,29], and the Electronic Medical Records and GEnomics (eMERGE) consortium [30].

Data are not shared as widely under this model, and large consortia have challenges, some of which are in common with the challenges of data sharing through dbGaP. The experiences of consortia with their internal data sharing and authorship policies may vary widely, and enforcement of those policies may be delicate because of relationships among collaborators. Often (but not always) large consortia do not have dedicated research funding to support additional data analysts, data coordinating centers, or common genotyping core facilities. The activities of the consortium must be conducted using resources on existing, potentially smaller-budget grants. Data transfer within consortia may also have logistical difficulties, such as the amount of time for transferring large scale sequencing data or the need for pre-processing and standardization, that are similar to the challenges for data sharing through dbGaP.

However, there are advantages of the consortium model. First, the research teams are working together, so any questions about how the data were collected or how phenotypes were defined can be resolved. Second, collaborating groups work directly with the IRBs of the institutions where the data were collected. In contrast, dbGaP data requestors frequently obtain IRB approval from their local IRB rather than the IRB responsible for the approval of the study for collecting data. Consortia deal with this issue directly by writing memoranda of understanding, and additionally, investigators add each other to their IRB protocols.. Third, as discussed above, there is direct acknowledgment of the data collectors via co-authorship. One recommendation to further enhance collaboration in the same spirit of the NIH data sharing policy is to expand research funding for consortia to support the activities of research teams. Indeed, some survey respondents implied support for this model when answering the open-ended questions throughout the survey. Further, the consortium model could be strengthened by developing a mechanism, perhaps through dbGaP, to publicize information about existing consortia so that interested researchers could contact members of those consortia to develop collaboration.

One reason for the development of dbGaP was to make data available for the purposes of methodology development. The Genetic Analysis Workshop (GAW) is another enormously successful model to stimulate collaboration among scientists interested in methodological questions. The GAW addresses questions about the robustness of analytical approaches for genetic data and serves as a forum for discussing new methods of analysis. The GAW has been ongoing since 1982 [31], and is supported through a grant from the National Institute of General Medical Sciences. The most recent GAW meeting in 2012 included 184 participants from 14 countries [32]. For each meeting, specific datasets are selected for analysis by an advisory committee, and are distributed to interested researchers about six months prior to the meeting. The workshop paradigm is particularly suited to advances in methodology development since all individuals are working on the same dataset(s), focusing on the same set of methodological questions, and meeting together to discuss results and learn from one another. Thus, we recommend additional funding for similar workshops.

### 4.4. Implications for Future Studies

The issues that have been identified for sharing GWAS data through dbGaP may be amplified when sharing WGS data. WGS datafiles are vastly larger than those from GWAS. Genetic epidemiologists have encountered problems in downloading 1000 Genomes data, and such problems will be intensified many-fold with deep coverage sequence data. Thus, effective sharing of sequence data may be limited to individuals with considerable computational resources, although this may change as computational technology advances. In addition, there is an ongoing debate about the identifiability of subjects from GWAS, WES or WGS data, and these concerns were raised by our survey respondents. Malin *et al.* [33] detail the conditions for identifiability, which include whether the data are “distinguishing” (genome data fall into this category) and the availability of a “naming resource”. Thus, the risks of identifiability may vary widely in human genetic studies and should be considered as these large datasets are made available. Furthermore, while clinically relevant information in GWAS data is limited to sex chromosome anomalies and a small number of disease risk SNPs [34], an added complexity of WGS data is the significantly greater potential of discovering “incidental findings” of well-characterized risk variants [2,34,35,36]. If in the original consent form the duty to report incidental findings is mentioned, complying with this obligation may be challenging when secondary data analysts make these discoveries. As the technology to create WGS data moves forward, there will be a great need to develop statistical methodologies to analyze these data, so the motivation to share these data is high in spite of the logistical concerns. Further discussion of the ethical implications of identifiability and incidental findings must precede the development of policies for sharing WGS data.

### 4.5. Limitations

There are some limitations of this survey and its analysis. First, the survey was only administered to IGES members, and thus may not be generalizable to all users of dbGaP. Geneticists with other specializations also have accessed or contributed to dbGaP, and so our results may not reflect their experiences. Second, the response rate to the survey was quite low (27% of IGES members), and it is unknown why a larger percentage of the IGES membership did not respond to the survey. Because of the low response rate, the study findings are susceptible to bias if individuals with particularly good or bad experiences preferentially responded to the survey. Also, we did not pilot test the survey questions with external reviewers to assess the readability or comprehension of the survey questions, so we do not know whether or not some respondents had difficulties responding to the questions. Finally, there are a number of topics that were not included in our survey, such as experiences with consortia, how long it takes to complete specific aspects of the application process including identifying the signing official, how data recipients used the data, and what were the perceived benefits of the policy.

## References

[1] NOT-OD-07-088. http://grants.nih.gov/grants/guide/notice-files/NOT-OD-07-088.html.

[2] Kaye J., Heeney C., Hawkins N., de Vries J., Boddington P. (2009). Data sharing in genomics—Re-shaping scientific practice. Nat. Rev. Genet..

[3] Guttmacher A.E., Nabel E.G., Collins F.S. (2009). Why data-sharing policies matter. Proc. Natl. Acad. Sci. USA.

[4] Homer N., Szelinger S., Redman M., Duggan D., Tembe W., Muehling J., Pearson J.V., Stephan D.A., Nelson S.F., Craig D.W. (2008). Resolving individuals contributing trace amounts of DNA to highly complex mixtures using high-density SNP genotyping microarrays. PLoS Genet..

[5] Jacobs K.B., Yeager M., Wacholder S., Craig D., Kraft P., Hunter D.J., Paschal J., Manolio T.A., Tucker M., Hoover R.N. (2009). A new statistic and its power to infer membership in a genome-wide association study using genotype frequencies. Nat. Genet..

[6] Clayton D. (2010). On inferring presence of an individual in a mixture: A Bayesian approach. Biostatistics.

[7] Gitschier J. (2009). Inferential genotyping of Y chromosomes in Latter-Day Saints founders and comparison to Utah samples in the HapMap project. Am. J. Hum. Genet..

[8] Nyholt D.R., Yu C.E., Visscher P.M. (2009). On Jim Watson’s APOE status: Genetic information is hard to hide. Eur. J. Hum. Genet..

[9] Gymrek M., McGuire A.L., Golan D., Halperin E., Erlich Y. (2013). Identifying personal genomes by surname inference. Science.

[10] Mascalzoni D., Janssens A.C., Stewart A., Pramstaller P., Gyllensten U., Rudan I., van Duijn C.M., Wilson J.F., Campbell H., Quillan R.M. (2010). Comparison of participant information and informed consent forms of five European studies in genetic isolated populations. Eur. J. Hum. Genet..

[11] Schekman R. (2009). PNAS takes action regarding breach of NIH embargo policy on a PNAS paper. Proc. Natl. Acad. Sci. USA.

[12] International Genetic Epidemiology Society about IGES. http://geneticepi.org/content/about-iges.

[13] SurveyMonkey. http://www.surveymonkey.com.

[14] Krawczak M., Goebel J.W., Cooper D.N. (2010). Is the NIH policy for sharing GWAS data running the risk of being counterproductive?. Investig Genet..

[15] It’s not about the Data. http://www.nature.com/ng/journal/v44/n2/full/ng.1099.html.

[16] Office for Human Research Protections, U.S. Department of Health & Human Services Title 45 Public Welfare Part 46 Protection of Human Subjects. http://www.hhs.gov/ohrp/humansubjects/commonrule/index.html.

[17] McGuire A.L., Gibbs R.A. (2006). Genetics. No longer de-identified. Science.

[18] McWilliams R., Hoover-Fong J., Hamosh A., Beck S., Beaty T., Cutting G. (2003). Problematic variation in local institutional review of a multicenter genetic epidemiology study. JAMA.

[19] Lemke A.A., Trinidad S.B., Edwards K.L., Starks H., Wiesner G.L. (2010). Attitudes toward genetic research review: Results from a national survey of professionals involved in human subjects protection. J. Empir Res. Hum. Res. Ethics.

[20] International Genetic Epidemiology Society Position Statement of the International Genetic Epidemiology Society in Response to “Draft NIH Genomic Data Sharing Policy Request for Public Comments”. http://www.geneticepi.org/wp-content/uploads/2013/11/IGES_Response_to_GDS_Policy_2013.pdf.

[21] Heard-Costa N.L., Zillikens M.C., Monda K.L., Johansson A., Harris T.B., Fu M., Haritunians T., Feitosa M.F., Aspelund T., Eiriksdottir G. (2009). NRXN3 is a novel locus for waist circumference: A genome-wide association study from the CHARGE Consortium. PLoS Genet..

[22] Fox E.R., Young J.H., Li Y., Dreisbach A.W., Keating B.J., Musani S.K., Liu K., Morrison A.C., Ganesh S., Kutlar A. (2011). Association of genetic variation with systolic and diastolic blood pressure among African Americans: The Candidate Gene Association Resource study. Hum. Mol. Genet..

[23] Zhu X., Young J.H., Fox E., Keating B.J., Franceschini N., Kang S., Tayo B., Adeyemo A., Sun Y.V., Li Y. (2011). Combined admixture mapping and association analysis identifies a novel blood pressure genetic locus on 5p13: Contributions from the CARe consortium. Hum. Mol. Genet..

[24] Naj A.C., Jun G., Beecham G.W., Wang L.S., Vardarajan B.N., Buros J., Gallins P.J., Buxbaum J.D., Jarvik G.P., Crane P.K. (2011). Common variants at MS4A4/MS4A6E, CD2AP, CD33 and EPHA1 are associated with late-onset Alzheimer’s disease. Nat. Genet..

[25] Saccone N.L., Culverhouse R.C., Schwantes-An T.H., Cannon D.S., Chen X., Cichon S., Giegling I., Han S., Han Y., Keskitalo-Vuokko K. (2010). Multiple independent loci at chromosome 15q25.1 affect smoking quantity: A meta-analysis and comparison with lung cancer and COPD. PLoS Genet..

[26] Liu J.Z., Tozzi F., Waterworth D.M., Pillai S.G., Muglia P., Middleton L., Berrettini W., Knouff C.W., Yuan X., Waeber G. (2010). Meta-analysis and imputation refines the association of 15q25 with smoking quantity. Nat. Genet..

[27] Thorgeirsson T.E., Gudbjartsson D.F., Surakka I., Vink J.M., Amin N., Geller F., Sulem P., Rafnar T., Esko T., Walter S. (2010). Sequence variants at CHRNB3-CHRNA6 and CYP2A6 affect smoking behavior. Nat. Genet..

[28] Adrianto I., Wen F., Templeton A., Wiley G., King J.B., Lessard C.J., Bates J.S., Hu Y., Kelly J.A., Kaufman K.M. (2011). Association of a functional variant downstream of TNFAIP3 with systemic lupus erythematosus. Nat. Genet..

[29] Lessard C.J., Adrianto I., Kelly J.A., Kaufman K.M., Grundahl K.M., Adler A., Williams A.H., Gallant C.J., Anaya J.M., Bae S.C. (2011). Identification of a systemic lupus erythematosus susceptibility locus at 11p13 between PDHX and CD44 in a multiethnic study. Am. J. Hum. Genet..

[30] McCarty C.A., Chisholm R.L., Chute C.G., Kullo I.J., Jarvik G.P., Larson E.B., Li R., Masys D.R., Ritchie M.D., Roden D.M. (2011). The eMERGE Network: A consortium of biorepositories linked to electronic medical records data for conducting genomic studies. BMC Med. Genomics.

[31] Genetic Analysis Workshop. www.gaworkshop.org.

[32] Bickeböller H., Bailey J.N., Beyene J., Cantor R.M., Cordell H.J., Culverhouse R.C., Engelman C.D., Fardo D.W., Ghosh S., König I.R. (2014). Genetic Analysis Workshop 18: Methods and strategies for analyzing human sequence and phenotype data in members of extended pedigrees. BMC Proceedings.

[33] Malin B., Karp D., Scheuermann R.H. (2010). Technical and policy approaches to balancing patient privacy and data sharing in clinical and translational research. J. Investig Med..

[34] Johnson A.D., Bhimavarapu A., Benjamin E.J., Fox C., Levy D., Jarvik G.P., O’Donnell C.J. (2010). CLIA-tested genetic variants on commercial SNP arrays: Potential for incidental findings in genome-wide association studies. Genet. Med..

[35] Wolf S.M., Lawrenz F.P., Nelson C.A., Kahn J.P., Cho M.K., Clayton E.W., Fletcher J.G., Georgieff M.K., Hammerschmidt D., Hudson K. (2008). Managing incidental findings in human subjects research: Analysis and recommendations. J. Law Med. Ethics.

[36] Fabsitz R.R., McGuire A., Sharp R.R., Puggal M., Beskow L.M., Biesecker L.G., Bookman E., Burke W., Burchard E.G., Church G. (2010). Ethical and practical guidelines for reporting genetic research results to study participants: Updated guidelines from a National Heart, Lung, and Blood Institute working group. Circ. Cardiovasc. Genet..

